# Association of statin use in older people primary prevention group with risk of cardiovascular events and mortality: a systematic review and meta-analysis of observational studies

**DOI:** 10.1186/s12916-021-02009-1

**Published:** 2021-06-22

**Authors:** Kamal Awad, Maged Mohammed, Mahmoud Mohamed Zaki, Abdelrahman I. Abushouk, Gregory Y. H. Lip, Michael J. Blaha, Carl J. Lavie, Peter P. Toth, J. Wouter Jukema, Naveed Sattar, Maciej Banach, on behalf of the Lipid and Blood Pressure Meta-analysis Collaboration (LBPMC) Group and the International Lipid Expert Panel (ILEP)

**Affiliations:** 1 Faculty of Medicine, Zagazig University, Zagazig, Egypt; 2 Department of Cardiovascular Medicine, Heart and Vascular Institute, Cleveland Clinic Foundation, Cleveland, OH USA; 3 Liverpool Centre for Cardiovascular Science, University of Liverpool and Liverpool Heart & Chest Hospital, Liverpool, UK; 4 The Ciccarone Center for the Prevention of Cardiovascular Disease, Johns Hopkins School of Medicine, Baltimore, MD USA; 5 John Ochsner Heart and Vascular Institute, Ochsner Clinical School-the University of Queensland School of Medicine, New Orleans, LA USA; 6 Preventive Cardiology, CGH Medical Center, Sterling, IL USA; 7 Department of Cardiology, Leiden University Medical Center, Leiden, the Netherlands and Netherlands Heart Institute, Utrecht, the Netherlands; 8 Institute of Cardiovascular and Medical Sciences, University of Glasgow, Glasgow, UK; 9 Head Department of Hypertension, WAM University Hospital in Lodz, Medical University of Lodz (MUL), Lodz, Poland; 10 Polish Mother’s Memorial Hospital Research Institute (PMMHRI), Lodz, Poland; 11 Cardiovascular Research Centre, University of Zielona Gora, Zielona Gora, Poland

**Keywords:** Statins, Older, Primary prevention, Myocardial infarction, Mortality, Stroke

## Abstract

**Background:**

Current evidence from randomized controlled trials on statins for primary prevention of cardiovascular disease (CVD) in older people, especially those aged > 75 years, is still lacking. We conducted a systematic review and meta-analysis of observational studies to extend the current evidence about the association of statin use in older people primary prevention group with risk of CVD and mortality.

**Methods:**

PubMed, Scopus, and Embase were searched from inception until March 18, 2021. We included observational studies (cohort or nested case-control) that compared statin use vs non-use for primary prevention of CVD in older people aged ≥ 65 years; provided that each of them reported the risk estimate on at least one of the following primary outcomes: all cause-mortality, CVD death, myocardial infarction (MI), and stroke. Risk estimates of each relevant outcome were pooled as a hazard ratio (HR) with a 95% confidence interval (CI) using the random-effects meta-analysis model. The quality of the evidence was rated using the GRADE approach.

**Results:**

Ten observational studies (9 cohorts and one case-control study; *n* = 815,667) fulfilled our criteria. The overall combined estimate suggested that statin therapy was associated with a significantly lower risk of all-cause mortality (HR: 0.86 [95% CI 0.79 to 0.93]), CVD death (HR: 0.80 [95% CI 0.78 to 0.81]), and stroke (HR: 0.85 [95% CI 0.76 to 0.94]) and a non-significant association with risk of MI (HR 0.74 [95% CI 0.53 to 1.02]). The beneficial association of statins with the risk of all-cause mortality remained significant even at higher ages (> 75 years old; HR 0.88 [95% CI 0.81 to 0.96]) and in both men (HR: 0.75 [95% CI: 0.74 to 0.76]) and women (HR 0.85 [95% CI 0.72 to 0.99]). However, this association with the risk of all-cause mortality remained significant only in those with diabetes mellitus (DM) (HR 0.82 [95% CI 0.68 to 0.98]) but not in those without DM. The level of evidence of all the primary outcomes was rated as “very low.”

**Conclusions:**

Statin therapy in older people (aged ≥ 65 years) without CVD was associated with a 14%, 20%, and 15% lower risk of all-cause mortality, CVD death, and stroke, respectively. The beneficial association with the risk of all-cause mortality remained significant even at higher ages (> 75 years old), in both men and women, and in individuals with DM, but not in those without DM. These observational findings support the need for trials to test the benefits of statins in those above 75 years of age.

**Supplementary Information:**

The online version contains supplementary material available at 10.1186/s12916-021-02009-1.

## Background

Cardiovascular disease (CVD) is the major cause of mortality worldwide [1–3]. More than 80% of the overall CVD death occurs in older people (aged ≥ 65 years) [2, 4]. In 2015, people aged ≥ 65 years represented 8.5% (617.1 million) of the global population (7.3 billion) [5]. In 2030, this percentage is projected to reach 12% (1 billion) of the world population [5]. In Europe, it is projected that almost 25% of its population will be aged ≥ 65 years by 2030, higher than any of the other continents [6, 7]. Therefore, efforts at the prevention of CVD in older people are important and will influence global healthcare policies.

The consideration of statins for primary CVD prevention in older people represents a dilemma in clinical practice unlike the secondary prevention, which is well-established and supported by a level (A) evidence, according to the most recent European Society of Cardiology (ESC)/European Atherosclerosis Society (EAS) guidelines [4, 8]. Current evidence on the use of statins for primary prevention of CVD in older people (especially those aged > 75 years) is still lacking. Statin therapy for primary prevention in people aged > 75 years (at high risk) was supported by level (B) evidence and considered as a class IIb recommendation in the 2019 ESC/EAS guidelines on dyslipidemias [4]. The 2019 ESC/EAS guidelines advocate statins for primary prevention in older people aged ≤ 75 years (i.e., 65 to 75 years) as a class I recommendation, which is unlike the 2016 guidelines with a class ΙΙa recommendation and level (B) evidence [4, 9]. This change in recommendation class was based on an individual participant level-meta-analysis from 28 randomized controlled trials (RCTs) by the “Cholesterol Treatment Trialists’ Collaboration” [4, 10] that reported a significant 39% proportional reduction [rate ratio (RR) 0.61; 99% confidence interval (CI) 0.51 to 0.73] in major vascular events for every 1 mmol/L drop in low-density lipoprotein cholesterol (LDL-C) by statins (or more intensive statin therapy) in participants without vascular disease, aged > 65 and ≤ 70 years [10]. This beneficial effect of statins was statistically insignificant for primary prevention in participants aged > 70 years [10].

One important limitation in the previous results is reporting only the treatment effect on a composite outcome (i.e., major vascular events) without reporting the results of its component outcomes [e.g., coronary artery disease death, stroke, myocardial infarction (MI), or coronary revascularization] that greatly varies in their individual clinical importance [11]. Therefore, these results may be somewhat misleading. Moreover, other study-level meta-analyses did not report any significant effect of statins for primary prevention in older people on any of the components of the mentioned composite except on MI (and on stroke in one meta-analysis) [11–14]. As a result, this beneficial effect of statins on the major CVD events could be driven entirely by their effects only on MI and stroke.

Other limitations of the current evidence from RCTs include: (Ι) underrepresentation of the older people; most of the available data are from subgroup analyses, and (ΙΙ) relatively short follow-up durations; especially when evaluating the treatment effect on mortality and the potential development of some side effects such as cancer incidence and new onset diabetes mellitus (NODM) that may require longer durations for adequate assessment. Consequently, observational studies on statins for primary prevention in older people may extend the current limited evidence through their larger populations, longer durations of follow-up, and better clinical practice generalizability than the RCTs. To our knowledge, no former meta-analysis assessed such outcomes from observational studies.

To address this issue, we conducted a systematic review and meta-analysis of the observational studies to provide better evidence about the association of statin use in older people primary prevention group (especially those aged ≥ 75 years) with the risk of CVD and mortality.

## Methods

We designed this study according to the Meta-analysis of Observational Studies in Epidemiology (MOOSE) guidelines (Supplementary Table 1) [15]. This study protocol was not prospectively registered. Due to the design of the study, it did not need any Institutional Review Board approval or patient informed consent.

### Literature search strategy

An electronic literature search of PubMed, Scopus, and Embase was conducted, without any restriction filters, from inception until March 18, 2021. We used a combination of relevant keywords and Medical Subject Headings (MeSH) terms reported in Supplementary Table 2. To avoid missing any related study, we conducted a manual search of the bibliographies of the included studies and of selected relevant reviews.

### Study selection

After removing the duplicates by Endnote X7 (Thompson Reuter, CA, USA), two independent authors (MM and MZ) performed a two-step screening of the remaining articles. Firstly, title/abstract screening then, full-text screening according to the predefined inclusion/exclusion criteria. Disagreement was resolved by the opinion of a third author (KA).

Original studies were included if they met the following criteria: (1) being an observational study (cohort or nested case-control), (2) compared using a statin (atorvastatin, fluvastatin, lovastatin, pitavastatin, pravastatin, rosuvastatin, or simvastatin) with no statins, (3) being restricted to or included a subgroup of older people aged ≥ 65 years and without established CVD (coronary artery disease, cerebrovascular disease, and peripheral vascular disease), and (4) reported the risk estimate as a hazard ratio (HR), odds ratio (OR) with its 95% CI on at least one of the following outcomes: all cause-mortality, CVD death, MI, and stroke.

Exclusion criteria included any of the following: (1) RCTs, experimental studies, reviews, theses, and book chapters, (2) studies whose full-texts were not available or with non-English content, (3) studies that contained made-up data or were retracted by the journal, or (4) studies that missed any of the inclusion criteria.

### Data extraction and outcomes of interest

Using preformatted tables, two independent authors (MM, MZ) reviewed the included articles and extracted the following data: (1) first author’s name, (2) year of publication, (3) study location, (4) study design, (5) follow-up duration, (6) study population characteristics, and (7) data regarding the relevant outcomes (i.e., the most adjusted risk estimates along with their 95% CI). Disagreements were resolved upon the opinion of another author (KA).

Primary outcomes were risk estimates of all cause-mortality, CVD death, MI, and stroke. Secondary outcomes included risk estimates on NODM and cancer incidence.

If a study reported its data on the older people as multiple age cohorts, in which participants of the first age cohort who survived to the next target age could have been a part of multiple age cohorts, we only extracted data of the age cohort with the largest sample size to avoid overlapped data.

### Risk of bias assessment

Two reviewers (KA and MZ) independently used The Risk Of Bias In Non-randomized Studies of Interventions (ROBINS-I) tool for the risk of bias assessment of the included studies [16]. This tool includes seven domains and rates the overall risk of bias as low, moderate, serious, critical, or unclear. Disagreements were resolved by discussion.

### Quality of evidence assessment

The level of evidence of each primary outcome was rated as very low, low, moderate, or high quality using the Grading of Recommendation Assessment, Development and Evaluation (GRADE) approach [17]. This rating system is based on five domains to downgrade the evidence level as follows: risk of bias, imprecision, inconsistency, indirectness, and publication bias; and other three domains for upgrading the evidence level as follows: large effect size, dose-response gradient, and all residual confounding reducing an effect size [18, 19].

### Quantitative data synthesis

Odds ratios from nested case-control studies may be considered to be equivalent to HRs from cohort studies obtained by the Cox regression analysis [20, 21]. Therefore, the most adjusted risk estimates were pooled as a HR with a 95%CI in a meta-analysis model. Given the probable heterogeneity across the included observational studies, the random-effects model was used for the analysis.

Between-study heterogeneity was measured by I^2^ and Chi^2^ tests. Interpretation of these tests was done in accordance with the “Cochrane handbook for systematic reviews of interventions,” in which an alpha level (for Chi^2^ test) < 0.1 is considered as significant heterogeneity and the I^2^ test is read as follows: 0–40%, might not be important; 30–60%, may represent moderate heterogeneity; and 50–90%, may represent substantial heterogeneity [22].

To investigate the risk estimate across different older ages and to address the heterogeneity, a subgroup analysis (based on the availability of relevant data for the intended subgroups in the included studies) was conducted according to the age of the participants as follows: 65 to 75 years, ≥ 75 years, ≥ 80 years, and ≥ 85 years. Other subgroup analyses according to sex, presence of DM and risk of bias level were also conducted. Subgroups focused only on all-cause mortality outcome, which was sufficiently reported in most of the included studies.

To assess the impact of each study on the overall combined risk estimate (i.e., results robustness), we conducted “leave-one-out sensitivity analysis,” by omitting one study each time and repeating the analysis.

Potential publication bias was assessed by visual inspection of Begg’s funnel plot asymmetry and confirmed by Egger’s regression test [23]. Funnel plot asymmetry (if present) was corrected using the “trim and fill” approach by imputing a number of theoretically missing studies [24]. All analyses were done by MetaXL version 5.3 (add-in for meta-analysis in Microsoft Excel; www.epigear.com) and Comprehensive Meta-Analysis version 3 (Biostat, NJ, USA).

## Results

### Flow and characteristics of included studies

**Fig. 1:**
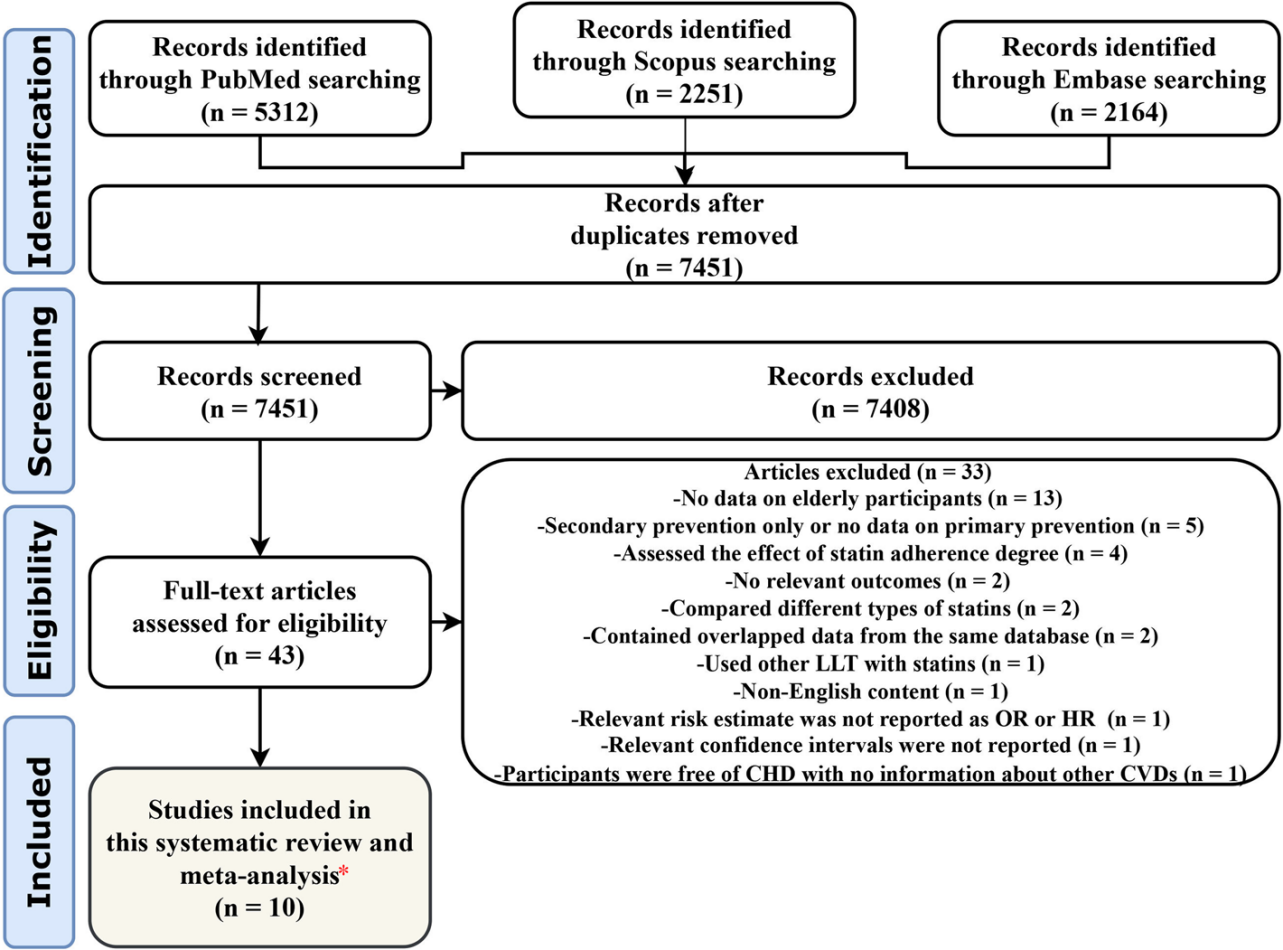
PRISMA flow diagram of study screening and selection. LLT, lipid-lowering treatment; OR, odds ratio; HR, hazard ratio; CHD, coronary heart disease; CVD, cardiovascular disease. *No additional eligible studies were found by manual search

The literature search yielded 9727 records. After removing the duplicates and the two-step screening, ten observational studies [25–34] with 815,667 relevant participants (without overlap) fulfilled our criteria and were included in this meta-analysis (see Fig. 1 for the PRISMA flow diagram). Supplementary Table 3 lists the excluded studies with reasons for exclusion.

Characteristics and baseline parameters of the included studies are shown in Table 1. Nine [25–31, 33, 34] of the included studies were cohort studies plus one [32] nested case-control study. The follow-up duration ranged from 4.7 to 24 years. The publication year ranged from 2002 to 2020. The included studies were conducted in Europe (*n* = 4), North America (USA; *n* = 4), and Asia (South Korea; *n* = 2). The covariates that were used for analysis adjustment in the included studies are shown in Supplementary Table 4.

**Table 1:** Characteristics and baseline parameters of the included studies

**Study**	**Year**			**Country**			**Design**			**Follow-up (years)**		**Population**				
Alpérovitch et al. [25]	2015			France			Prospective cohort			9.1^a^		Older people ≥ 65 years without a history of CVD				
Bezin et al. [26]	2019			France			Retrospective cohort			4.7^b^		People ≥ 75 years with and without a history of CVD				
Gitsels et al. [27]	2016			UK			Retrospective cohort			16–24		People aged 60, 65, 70, and 75 years without a history of CVD stratified according to the QRISK2 score				
Jun et al. [32]	2019			South Korea			Nested case-control			NA		People who developed first time CV event or death ≥ 75 years and their matched controls				
Kim et al. [28]	2019			South Korea			Retrospective cohort			5.2^b^		Patients > 75 years with at least one CV risk factor (HTN, DM, or overweight) and without a history of CVD				
Lemaitre al [31].	2002			USA			Prospective cohort			Up to 7.3		Older people ≥ 65 years without a history of CVD				
Orkaby et al. [29]	2017			USA			Prospective cohort			7^b^		Male physicians ≥ 70 years without a history of CVD				
Orkaby et al. [33]	2020			USA			Retrospective cohort			6.8^a^		US veterans ≥ 75 years without history of CVD				
Ramos et al. [30]	2018			Spain			Retrospective cohort			5.6^b^		Older people ≥ 75 years without a history of CVD				
Zhou et al. [34]	2020			Australia, USA			Retrospective cohort			4.7^b^		Older people from ASPREE trial ≥ 70 years without a history of CVD, dementia, and physical disability				
**Study**	**Groups of interest**		**Age, (years)**	**Women**	**BMI**	**LDL-C**	**HDL-C**	**TG**	**Family history of CVD**	**Smoker**		**Alcoholic**		**DM**	**HTN**	**Renal disease**
										**EX**	**Current**	**Ex**	**Current**			
Alpérovitch et al. [25]	Statin prevalent users (*n* = 1007)		73.1 (4.6)	67.8	25.8 (4.0)	3.40 (0.9)	1.64 (0.4)	1.27 (0.84–1.93)^c^	NR	30.2	4.4	1.1	81.8	10.9	79.7	NR
	No LLT (*n* = 5436)		74.1 (5.6)	62	25.4 (4.0)	3.78 (0.9)	1.63 (0.4)	1.14 (0.76–1.70)^c^	NR	31.3	6.3	2.8	79.7	7.2	74.5	NR
Bezin et al. [26]	Primary prevention without modifiable risk factors (*n* = 752)		78 (76–81)^c^	71.8	NR	NR	NR	NR	NR	NR	NR	NR	NR	0	0^d^	NR
Gitsels et al. [27]	QRISK2 < 10%	Statin prevalent users (*n* = 883)	65	100	26 (4)	NR	NR	NR	0	10	0	NR	NR	0	5	0
		No LLT (*n* = 39,866)	65	100	26 (4)	NR	NR	NR	0	10	3	NR	NR	0	1	0
		Statin prevalent users (*n* = 3)	70	100	28 (6)	NR	NR	NR	0	0	0	NR	NR	0	0	0
		No LLT (*n* = 322)	70	100	25 (4)	NR	NR	NR	0	4	3	NR	NR	0	0	0
	QRISK2 = 10–19%	Statin prevalent users (*n* = 6438)	65	68	28 (5)	NR	NR	NR	1	24	10	NR	NR	7	56	0
		No LLT (*n* = 116,240)	65	47	26 (4)	NR	NR	NR	0	21	20	NR	NR	1	24	0
		Statin prevalent users (*n* = 10,822)	70	92	27 (5)	NR	NR	NR	1	20	1	NR	NR	0	55	2
		No LLT (*n* = 108,703)	70	86	26 (5)	NR	NR	NR	0	17	5	NR	NR	0	21	0
		Statin prevalent users (*n* = 661)	75	100	26 (4)	NR	NR	NR	0	5	0	NR	NR	0	2	0
		No LLT (*n* = 13,685)	75	100	25 (4)	NR	NR	NR	0	5	1	NR	NR	0	0	0
	QRISK2 ≥ 20%	Statin prevalent users (*n* = 5259)	65	33	29 (5)	NR	NR	NR	1	32	32	NR	NR	59	77	0
		No LLT (*n* = 29,170)	65	16	27 (5)	NR	NR	NR	1	19	57	NR	NR	22	49	0
		Statin prevalent users (*n* = 25,559)	70	37	29 (5)	NR	NR	NR	2	38	18	NR	NR	39	73	12
		No LLT (*n* = 98,900)	70	24	26 (4)	NR	NR	NR	1	28	31	NR	NR	8	37	2
		Statin prevalent users (*n* = 34,743)	75	56	28 (5)	NR	NR	NR	1	34	10	NR	NR	29	74	15
		No LLT (*n* = 142,521)	75	55	26 (4)	NR	NR	NR	0	25	16	NR	NR	5	39	2
Jun et al. [32]	Cases (*n* = 11,017)		83.7 (3.2)	63.2	NR	NR	NR	NR	NR	NR	NR	NR	NR	14.7	44.2	NR
	Controls (*n* = 55,085)		83.7 (3.2)	63.2	NR	NR	NR	NR	NR	NR	NR	NR	NR	11.5	49.9	NR
Kim et al. [28]	Statin new users (*n* = 639)		78 (76–80)^c^	64.6	23.4 (22.2–25.8)^c^	107 (85–133)^c^	45 (39–54)^c^	110 (82–150)^c^	NR	NR	NR	NR	NR	32.6	95.6	3.1
	No statin (*n* = 639)		78 (76-80)^c^	61.3	23.3 (22–25.6)^c^	107 (85–129)^c^	46 (38–55)^c^	107 (79–151)^c^	NR	NR	NR	NR	NR	30.8	95.9	3.1
Lemaitre al [31].	Treated prevalent users (*n* = 251)		71.1 (4.6)	68.5	26.9 (4.4)	142.7 (42.2)	53.6 (15.8)	154.2 (87.1)	35.3	NR	9.6	NR	49.4	21.9	48.2	NR
	Untreated	Drug Recommended (*n* = 717)	72.7 (5.6)	66.7	27.4 (4.5)	177.2 (28.8)	50.9 (12.1)	153.1 (56.5)	42.5	NR	14.6	NR	45.3	20.5	48.1	NR
		Diet Recommended (*n* = 946)	72.5 (5.3)	63.4	27.2 (5)	147.5 (21)	53.0 (13.7)	141.4 (57)	36.4	NR	13.9	NR	48.9	20	43.7	NR
Orkaby et al. [29]	Statin prevalent users (*n* = 1130)		76 (4.5)	0	25.6 (3.1)	NR	NR	NR	NR	48.9	2.9	NR	NR	13	73.8	10.8
	No statin (*n* = 1130)		76 (4.6)	0	25.6 (3.2)	NR	NR	NR	NR	50.5	3.3	NR	NR	13.1	75.3	10.6
Orkaby et al. [33]	Statin new users (*n* = 57,178)		81.2 (3.6)	2.7	27.5 (4.3)	NR	NR	NR	NR	63.5	7.4	NR	NR	27	80.4	2.3
	No statin (*n* = 326,981)		80.7 (4.0)	2.7	26.7 (4.4)	NR	NR	NR	NR	71.9	7.3	NR	NR	13.1	66.2	1.1
Ramos et al. [30]	No T2DM, 75–84 years.	Statin new users (*n* = 4802)	78.8 (2.7)	65.1	28.6 (4.6)	3.9 (1.0)	1.5 (0.4)	1.4 (0.7)	NR	NR	13.5	NR	NR	0	65.7	NR
		No statin (*n* = 27,114)	79.1 (2.8)	62.8	28.4 (4.6)	3.3 (0.7)	1.5 (0.4)	1.2 (0.5)	NR	NR	12.4	NR	NR	0	57.3	NR
	No T2DM, ≥ 85 years.	Statin new users (*n* = 743)	88.5 (3.2)	69.8	27.1 (4.3)	3.7 (1.0)	1.5 (0.4)	1.4 (0.6)	NR	NR	7.8	NR	NR	0	66.8	NR
		No statin (*n* = 6325)	88.6 (3.2)	69.8	27.6 (4.5)	3.1 (0.8)	1.6 (0.4)	1.2 (0.5)	NR	NR	6.7	NR	NR	0	58.7	NR
	T2DM, 75–84 years	Statin new users (*n* = 1756)	78.8 (2.6)	61.3	29.7 (4.7)	3.7 (0.9)	1.4 (0.4)	1.7 (0.8)	NR	NR	15.4	NR	NR	100	78.4	NR
		No statin (*n* = 4885)	79.2 (2.8)	58	29.4 (4.8)	3 (0.7)	1.4 (0.4)	1.4 (0.7)	NR	NR	14.7	NR	NR	100	75.1	NR
	T2DM, ≥ 85 years	Statin new users (*n* = 201)	88.2 (2.8)	67.2	28.2 (4.3)	3.3 (1.0)	1.4 (0.3)	1.6 (0.9)	NR	NR	6.5	NR	NR	100	82.6	NR
		No statin (*n* = 1038)	88.2 (2.7)	68	27.5 (4.4)	3 (0.7)	1.4 (0.4)	1.4 (0.7)	NR	NR	8.2	NR	NR	100	75.8	NR
Zhou et al. [34]	Statin prevalent users (*n* = 5629)		74.2 (71.8–77.7)^c^	60.6	NR	NR	NR	NR	65.1	41.6	3.8	NR	75.6	19.6	82.4	29.9
	No statin (*n* = 12,467)		74.2 (71.8–77.9)^c^	54.0	NR	NR	NR	NR	59.3	40.6	3.4	NR	78.3	6.1	70.8	24.0

Continuous data are presented as mean (standard deviation)

Dichotomous data are presented as percentage

Abbreviations: *CVD* cardiovascular disease, *HTN* hypertension, *DM* diabetes mellitus, *BMI* body mass index, *LDL-C* low-density lipoprotein cholesterol, *HDL-C* high-density lipoprotein cholesterol, *TG* triglycerides, *LLT* lipid-lowering treatment, *NR* not reported, *ASPREE* Aspirin in Reducing Events in the Elderly

^a^Data are presented as mean

^b^Data are presented as median

^c^Data are presented as median (interquartile range)

^d^Participants on antihypertensive drugs

### Risk of bias in the included studies

According to the ROBINS-I tool, the risk of bias was rated as moderate in four included studies and as serious in six studies. One important source of the serious risk of bias in six included studies was including prevalent statin users (i.e., who initiated statin therapy prior to their inclusion in the study) instead of statins new users (i.e., who initiated statin therapy at their inclusion in the study). The risk of bias assessment is summarized in Supplementary Table 5.

### Outcome overall analysis

#### Primary outcomes

**Fig. 2:**
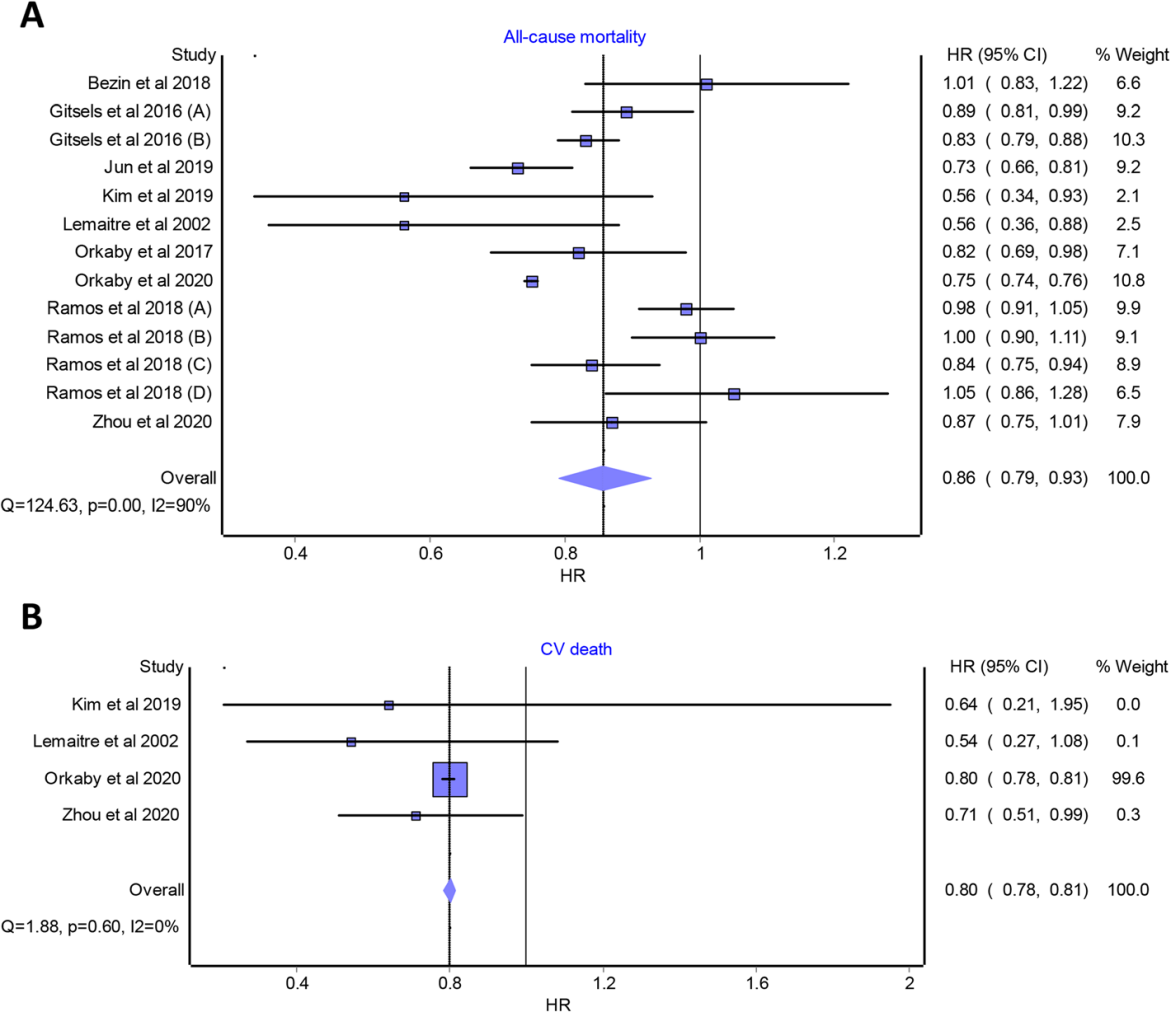
Forest plots displaying the results of the meta-analysis of observational studies that compared statin use with non-use in older people aged ≥ 65 years and without cardiovascular disease—**A** in terms of all-cause mortality and **B** in terms of cardiovascular death. HR, hazard ratio; CI, confidence interval; CV, cardiovascular

**Fig. 3:**
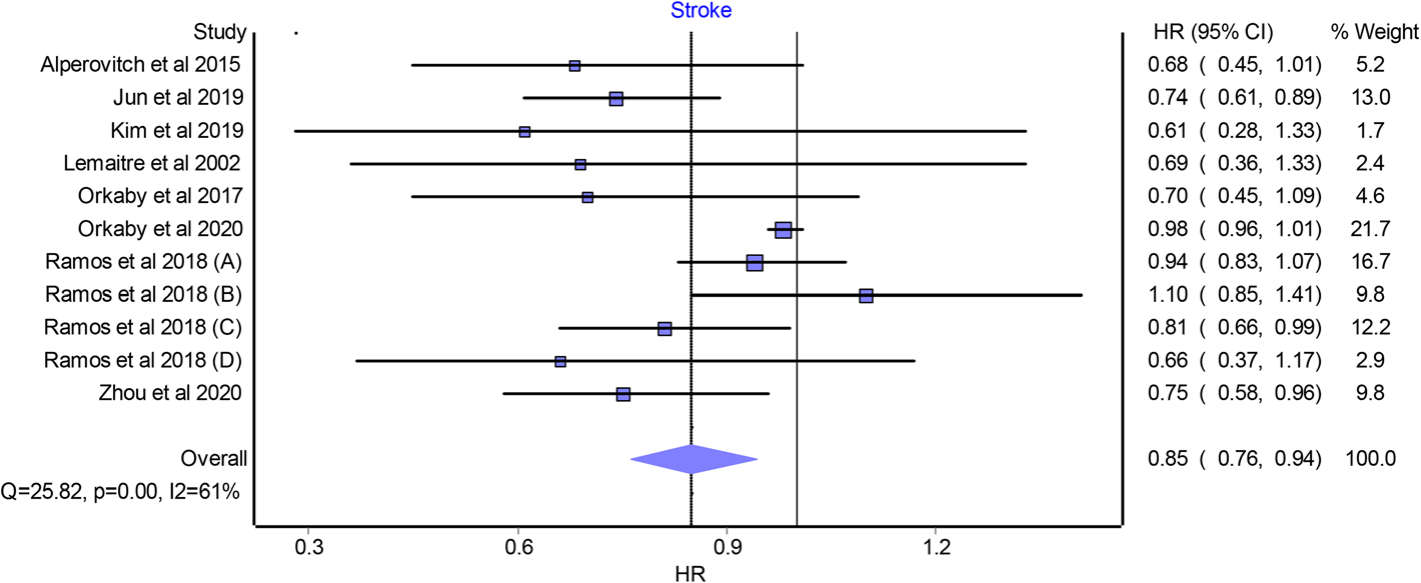
Forest plot displaying the results of the meta-analysis of observational studies that compared statin use with non-use in older people aged ≥ 65 years and without cardiovascular disease in terms of stoke. HR, hazard ratio; CI, confidence interval

**Fig. 4:**
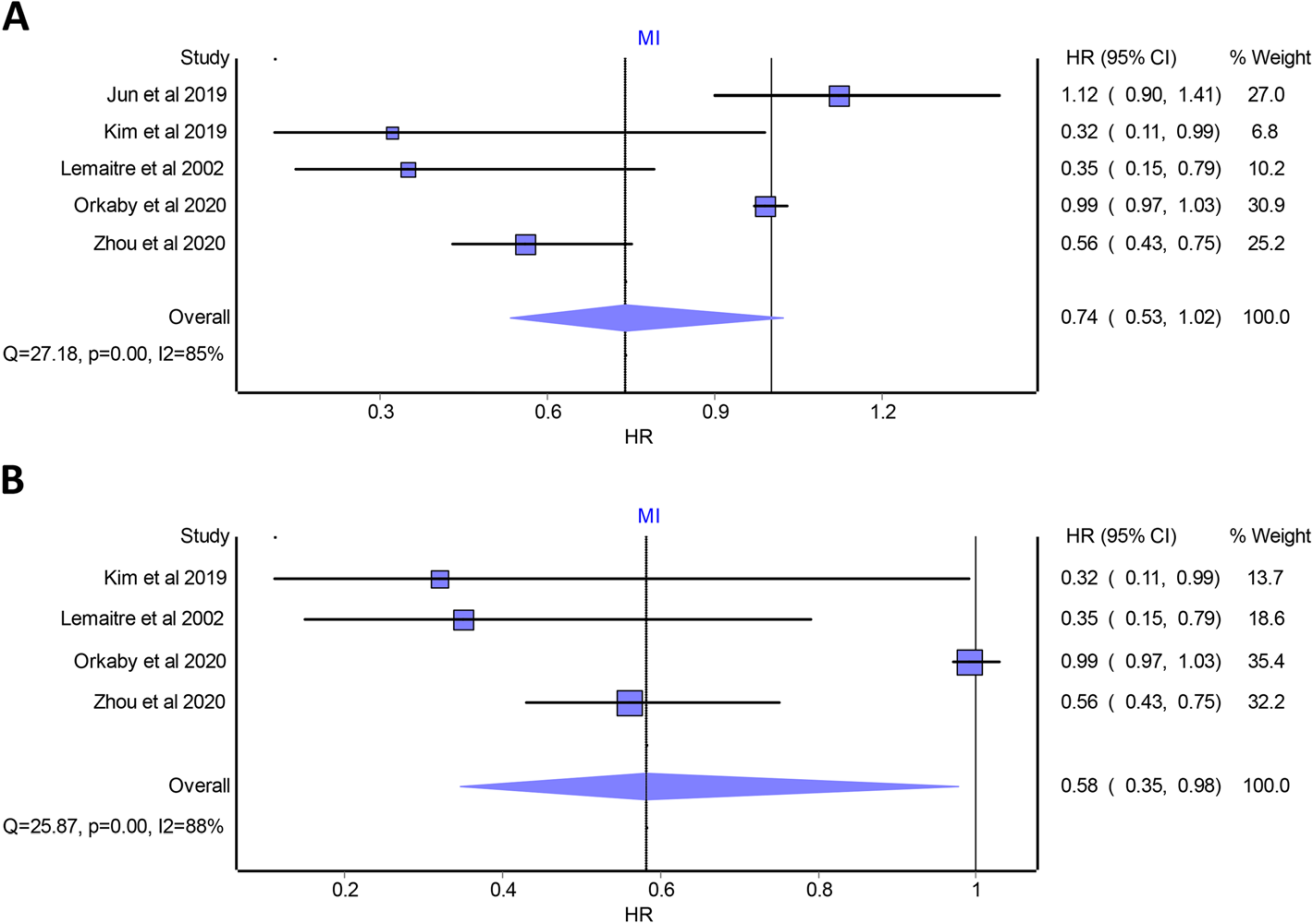
Forest plots displaying the results of the meta-analysis of observational studies that compared statin use with non-use in older people aged ≥ 65 years and without cardiovascular disease in terms of myocardial infarction—**A** before removing the study by Jun et al. and **B** after removing the study by Jun et al. HR, hazard ratio; CI, confidence interval; MI, myocardial infarction

Overall pooled analysis suggested that statin use was significantly associated with a lower risk of all-cause mortality (HR 0.86 [95% CI 0.79 to 0.93]; studies *n* = 9; Fig. 2A), CVD death (HR 0.80 [95% CI 0.78 to 0.81]; studies *n* = 4; Fig. 2B), and stroke (HR 0.85 [95% CI 0.76 to 0.94]; studies *n* = 8; Fig. 3) compared with statin non-use in the included population. A non-significant association was found between statin use and the risk of MI (HR 0.74 [95% CI 0.53 to 1.02]; studies *n* = 5; Fig. 4A). A significant heterogeneity was observed between the included studies in case of all-cause mortality (I^2^ = 90%, *P* value < 0.0001), stroke (I^2^ = 61%, *P* value < 0.0001), and MI (I^2^ = 85%, *P* value < 0.0001). No heterogeneity was observed in the case of CVD death (I^2^ = 0%, *P* value = 0.6). According to the GRADE approach, the level of evidence of all the primary outcomes was rated as “very low.” The quality of evidence assessment is summarized in Supplementary Table 6.

#### Secondary outcomes

There was a non-significant association between statin use and the risk of T2DM (HR 0.90 [95% CI 0.72 to 1.12]; studies *n* = 2; Supplementary Figure 1A) or new-onset cancer (HR 1 [95% CI 0.94 to 1.06]; studies *n* = 3; Supplementary Figure 1B) compared with statin non-use. No significant heterogeneity was observed in the case of both outcomes (*P* value > 0.1).

### Subgroup analysis

**Fig. 5:**
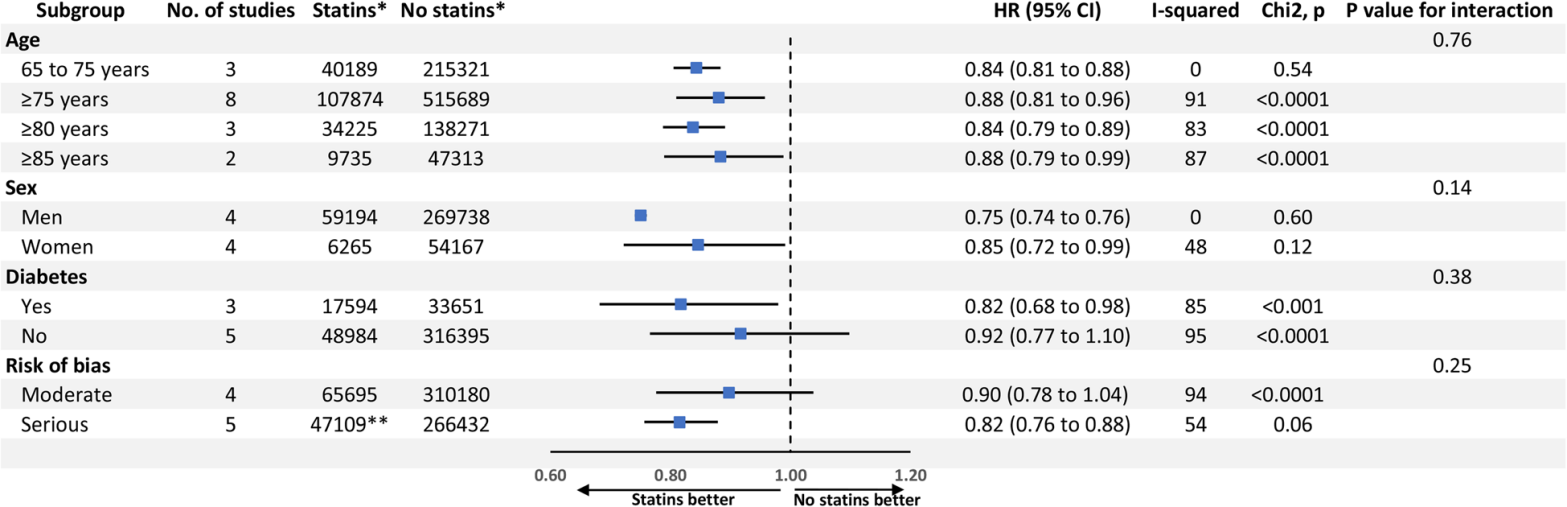
Forest plot displaying the results of the subgroup analysis (according to age, sex, diabetes mellitus, and risk of bias) of observational studies that compared statin use with non-use in older people aged ≥ 65 years and without cardiovascular disease in terms of all-cause mortality. HR, hazard ratio; HCI, higher confidence interval; LCI, lower confidence interval. *Number of included participants. **The exact number of statin users in the study by Lemaitre et al. was not reported and not added to the presented number

Subgroup analysis suggested that statin use was significantly associated with a lower risk of all-cause mortality, compared with statin non-use, in all age subgroups as follows: 65 to 75 years (HR 0.84 [95% CI 0.81 to 0.88]; studies *n* = 3, Fig. 5), ≥ 75 years (HR 0.88 [95% CI 0.81 to 0.96]; studies *n* = 8; Fig. 5), ≥ 80 years (HR 0.84 [95% CI 0.79 to 0.89]; studies *n* = 3; Fig. 5), and ≥ 85 years (HR 0.88 [95% CI 0.79 to 0.99]; studies *n* = 2; Fig. 5). Heterogeneity became insignificant in case of “65 to 75 years” subgroup (I^2^ = 0%, *P* value = 0.57). There was still a significant heterogeneity in the other age subgroups (*P* value < 0.0001).

In the subgroup analysis according to sex, statin use was significantly associated with a lower risk of all-cause mortality, compared with statin non-use, in both men (HR 0.75 [95% CI 0.74 to 0.76]; studies *n* = 4; Fig. 5) and women (HR 0.85 [95% CI 0.72 to 0.99]; studies *n* = 4; Fig. 5). No significant heterogeneity was observed in both subgroups (*P* value > 0.1).

As for subgroups according to DM, a significant association was found between statin use and the risk of all-cause mortality only in older people with DM (HR 0.82 [95% CI 0.68 to 0.98]; studies *n* = 3; Fig. 5) but not in those without DM (HR 0.92 [95% CI 0.77 to 1.10]; studies *n* = 5; Fig. 5). A significant heterogeneity was observed in both subgroups (*P* value < 0.0001).

In the subgroup analysis according to risk of bias level within the included studies, statin use was significantly associated with a lower risk of all-cause mortality, compared with statin non-use, only in case of serious risk of bias (HR 0.82 [95% CI 0.76 to 0.88]; studies *n* = 5; Fig. 5) but not in case of moderate risk of bias (HR 0.90 [95% CI 0.78 to 1.04]; studies *n*=4; Fig. 5). A significant heterogeneity was observed in both subgroups (*P* value < 0.1).

### Sensitivity analysis

The “leave-one-out” sensitivity analysis suggested that all overall combined risk estimates were robust except for MI outcome. As previously stated, the overall risk estimate for MI was as follows: (HR 0.74 [95% CI 0.53 to 1.02]; studies *n* = 5; Fig. 4A). After omitting the study by Jun et al. [32], this overall risk estimate became as follows: (HR 0.58 [95% CI 0.35 to 0.98]; studies *n* = 4; Fig. 4B), indicating a significant lower risk of MI with statin use compared with statin non-use.

### Publication bias

Visual inspection of funnel plots asymmetry indicated a potential publication bias in terms of all relevant outcomes (Supplementary Figure 2). The effect estimates of the outcomes were corrected using the “trim and fill” method by imputing 1–4 hypothetically missing studies (for each outcome). The significance/insignificance of the effect estimates was not altered after the adjustment for all outcomes (Supplementary Table 7). Egger’s test suggested a potential publication bias only in terms of all-cause mortality (*P* value = 0.026), stroke (*P* value = 0.003), and new-onset cancer (*P* value = 0.042) and excluded the presence of publication bias for the rest of the outcomes (*P* value > 0.05; Supplementary Table 7).

## Discussion

This meta-analysis suggests that statin therapy may be associated with a significant lower risk of all-cause mortality, CVD death, and stroke in older people aged ≥ 65 years without CVD. The beneficial association of statins with the risk of all-cause mortality remained significant even at higher ages. It also was significant in both men and women. The association with all-cause mortality remained significant only in older people with DM but not in those without DM. A non-significant association was found between statins and MI, and this issue requires further investigation.

These results are of interest especially in the context of the involved mechanism(s). Several epidemiological studies revealed no association or even an inverse association between low total cholesterol (TC), specifically LDL-C, and all-cause mortality in older people [35]. However, this paradox could be explained by some endogenous factors that may affect both TC and mortality, indicating reverse causation [36, 37]. One of these factors is “terminal decline”; in a cohort study with 99,758 participants aged 80 to 105 years, Charlton et al. [38] found a greater decline in TC levels in the last 2 years of life. Another factor is that low TC level was found to be a pre-diagnostic marker of several types of cancer [39, 40]. In addition, inflammation can reduce the serum LDL-C level through increasing the LDL receptor expression on the hepatocytes [41–44]. This response is mediated by the elevated cytokines; interleukin (IL)-1β, IL-6, tumor necrosis factor-α, and others [45, 46]. In this context, a recent meta-analysis of nine prospective studies (*n* = 9087 participants) revealed that high levels of IL-6 were associated with a higher risk of all-cause mortality in older people [47]. In line with these possible explanations, Mendelian randomization found that elevated LDL-C still carries a higher risk of mortality even in the oldest old people (> 90 years) [48].

In a meta-analysis that included eight RCTs, Savarese et al. [12] found that statins significantly reduce the risk of stroke (RR 0.76 [95% CI 0.63 to 0.93], I^2^ = 43.7%, studies *n* = 5) compared with placebo in older participants without CVD. Our finding on stroke is in line with this meta-analysis. In contrast, a recent meta-analysis including nine RCTs conducted by Ponce et al. [14] suggested no benefit of statins on stroke prevention (RR 0.78 [95% CI 0.6 to 1.01], I^2^ = 58.1%, studies *n* = 6), compared with placebo, in older participants without CVD. However, the result of this meta-analysis [14], unlike Savarese et al. [12], was not robust according to the sensitivity analysis which was statistically significant (RR 0.51, 95% CI 0.33 to 0.78) when the analysis was limited to studies that only included older participants or had planned for a subgroup analysis according to age.

In contradistinction to the aforementioned meta-analyses of RCTs [12, 14], our study suggested a non-significant association between statin use and MI. This finding should be cautiously interpreted because the overall risk estimate of MI became significant after removing the study conducted by Jun et al. [32], which was the only included case-control study. Jun and colleagues explained their finding on MI by the possibility that most of the included participants could have received low- to moderate-intensity statins which are less effective than high-intensity statins for MI prevention [32].

Unlike the above-mentioned meta-analyses of RCTs [12, 14], our study suggested a beneficial association with statins in terms of all-cause mortality and CVD death. In a recent meta-analysis including 40 RCTs, Yebyo et al. [49] investigated the efficacy and safety of statins for primary prevention of CVD in 94,283 participants of a wide age range. They found that statins significantly reduced the risk of all-cause mortality (RR 0.89, 95% CI 0.85 to 0.93) in the included population. Interestingly, the observed association with all-cause mortality in our study (HR 0.86 [95% CI 0.79 to 0.93]) was similar to that in the meta-analysis by Yebyo et al. [49]. In a recent Bayesian analysis of available data on older people from 35 RCTs, Kostis et al. [50] reported that people aged > 75 years on statins for primary prevention may have a lower mortality (*p* = 0.03). In line with our study, statin use for primary prevention was also associated with a lower risk of all-cause mortality (HR 0.83; *p* = 0.04) compared with statin non-use in a retrospective cohort included 1370 older Korean adults (aged ≥ 75 years); this observed association was more evident (HR 0.76; *p* = 0.01) in case of statin use for more than 5 years [51]. However, our observational findings on all-cause mortality should be interpreted cautiously because, in clinical practice, older people with short life expectancy (e.g., with malignancy) are less likely to receive statins and this might have introduced bias into the observed results [52, 53]. Even so, the cumulating evidence seems to be broadly consistent. In the general population, statins seem to reduce all-cause mortality primarily by reducing CVD death, and this is in line with our significant result in terms of CVD death [49, 54]. Only one of the included studies reported the association of statins with the risk of non-vascular mortality [28]. In this study, Kim et al. [28] observed that non-CVD deaths were less frequent in the statin users than non-users, but this was statistically non-significant.

In the present study, the observed beneficial association of statins with the risk of all-cause mortality remained significant even at higher ages. This finding is in line with the previously mentioned result from Mendelian randomization studies about the preserved risk role of elevated LDL-C in the oldest old people (> 90 years) [48]. However, the subgroups of the highest ages (≥ 80 and ≥ 85 years) were derived from a small number of studies that included a relatively limited sample size of this population. According to the reported analysis based on DM presence, the beneficial association with statins in terms of all-cause mortality was only evident in participants with DM, which highlights the need to more use of statin therapy in older people with DM in primary prevention. The aforementioned finding is in line with data from the Age, Gene/Environment Susceptibility (AGES)-Reykjavik study [55]. That prospective cohort study included 5,152 participants with an age range of 66 to 96 years, showing that statins were associated with a lower risk of all-cause mortality in DM participants comparable with non-DM, and regardless of coronary heart disease or glucose-lowering therapy [55]. Diabetes mellitus is associated with a 2- to 4-fold higher risk of CVD events [56]. Moreover, patients with longstanding DM (≥ 10 years), and without CVD, may be comparable to coronary heart disease (CHD) patients without DM in terms of future CHD events [57, 58]. Therefore, the above-mentioned result is clinically plausible.

The beneficial association with statins in terms of all-cause mortality was still evident in the subgroup that included studies with serious risk of bias but not in that with moderate risk of bias studies. Inclusion of prevalent statin users instead of statin new users in all the studies with serious risk of bias, denoting the long-term effect of statins, may explain this observed result [59, 60]. However, other sources of bias (e.g., bias due to confounding) might have affected this result.

The older population has a higher risk of drugs adverse effects because of multiple comorbidities, polypharmacy, and altered pharmacokinetics and pharmacodynamics [61–66]. Statin safety in this population is a point of concern as statin-related adverse effects are the most common cause of statin discontinuation [67–69]. In a meta-analysis that included more than 3 million older subjects, only 47.9% of statin users were adherent to therapy after one year of follow-up for primary prevention [70]. In terms of safety, our study found no significant association between statin use and the risk of incident T2DM or new-onset cancer. These findings are in line with evidence from primary prevention RCTs of older people [12, 13, 71–74] and even of participants with a wide age range [49]. In contrast, evidence including general mixed population (i.e. primary and secondary CVD prevention) reported a 9 to 55% increased risk of T2DM in statin users compared with statin non-users [75–77]. In a recent meta-analysis/meta-regression with more than 4 million participants (statins vs no statins) aged ≥ 30 years, older participants were associated with a decreased risk of T2DM (RR 0.79 [95% CI 0.63–0.98] per 10-year older) compared with younger participants [76]. The reported increase in statin-associated T2DM risk is mainly evident in participants who already are at a high risk to acquire DM (e.g., people with other elements of the metabolic syndrome) [75, 78].

None of the included studies reported data on other statin safety concerns in older people, including statin-associated muscle symptoms (SAMS) or cognitive impairment except one study by Zhou et al. [34] that reported a nonsignificant association between statins and risk of dementia (HR 1.14 [95% CI 0.94 to 1.39]). Current evidence from individual RCTs [72, 73, 79–81] and meta-analyses [82–85] reported no increased risk of SAMS with statins in older people or of cognitive impairment in the general population. As for evidence from observational studies, a meta-analysis of 25 prospective cohorts of the cognitively healthy general population found that statin use was associated with a lower risk of all-cause dementia (mean age 59.3 years), mild cognitive impairment (mean age 68.4 years), and Alzheimer’s disease (mean age 71.3 years) but not of vascular dementia (mean age 77.2 years) [86]. In terms of SAMS, an observational study including 4355 participants aged ≥ 75 years from the Netherlands found no difference in the prevalence of self-reported muscle symptoms in statin users compared with statin non-users (OR 1.39; 95% CI 0.94 to 2.05) regardless of CVD history [87]. Table 2 shows a comparison between results from meta-analyses of RCTs and our study or other observational studies in older people for statins used in primary prevention.

**Table 2:**
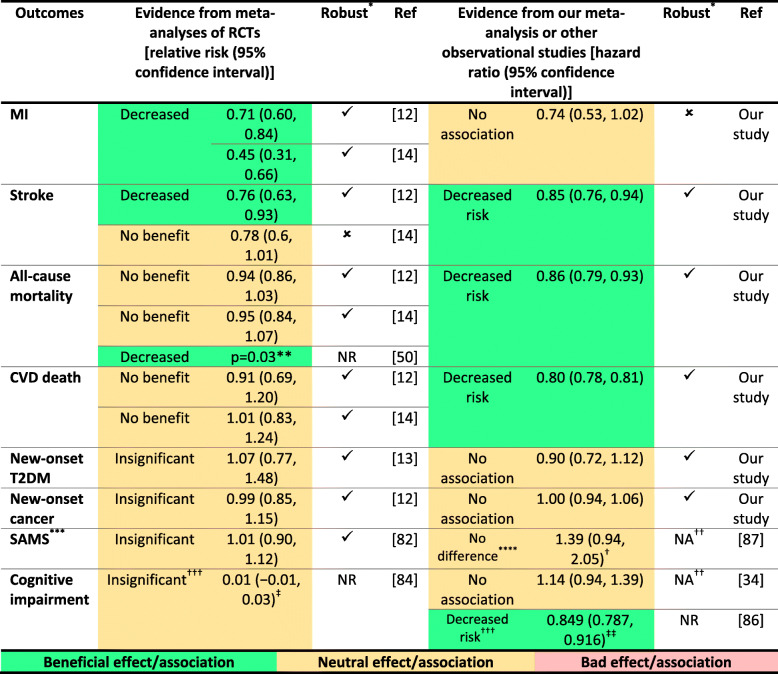
Summary of current evidence on statins for primary prevention in older people as a comparison between results from meta-analyses of RCTs and our study or other observational studies

Abbreviations: *RCTs* randomized controlled trials, *Ref* references, *MI* myocardial infarction, *CV* cardiovascular, *T2DM* type 2 diabetes mellitus, *SAMS* statin-associated muscle symptoms, *NA* not applicable, *NR* not reported

^*^According to sensitivity analysis

^**^Bayesian analysis

^***^Not reported in any of the included studies

^****^Regardless of cardiovascular disease history

^†^Data are reported as odds ratio (95% confidence interval)

^††^Not a meta-analysis

^†††^Data of the general population (including older participants not exclusively of older people) with normal cognition

^‡^Data are reported as standardized mean difference (95% confidence interval)

^‡‡^Data are reported as relative risk (95% confidence interval)

Older people are heterogenous in many aspects (i.e., clinically, demographically, and functionally) [36]. They were underrepresented in the available RCTs especially those aged ≥ 75 years, and this may limit the generalizability of their findings to the clinical practice. On the other hand, our study was based on data from observational studies, which are more generalizable. Another limitation of the evidence from older people RCTs is the relatively short follow-up durations that ranged from 1 to 5.2 years [12]. However, the follow-up durations of the included cohort studies in the current meta-analysis were longer (ranged from 4.7 to 24 years). Larger included number of participants, longer durations of follow-up, residual confounders, and inclusion of prevalent statin users instead of statin new users in some of the included observational studies are possible explanations for the observed differences between our study and RCTs in case of some outcomes (as presented above). Some previous meta-analyses of RCTs reported statins effect on composite outcomes of several components that significantly varies in their clinical importance (e.g. coronary revascularization versus CVD death) [10, 13]. It is recommended to avoid combining composite outcomes in meta-analyses to avoid any misleading results [88]. In our study, we only combined data of single components, and we filled some gaps in the evidence about people aged ≥ 75 years, while awaiting the results of The STAREE (STAtins for Reducing Events in the Elderly) trial (NCT02099123) and the PREVENTABLE (Pragmatic Evaluation of Events and Benefits of Lipid-Lowering in Older Adults) trial (NCT04262206).

### Limitations

This meta-analysis has several limitations. First, it is based only on observational studies. One critical source of bias in six included studies was including prevalent statin users instead of statins new users. Thus, the present results might have been influenced by residual confounding. Second, unresolved heterogeneity was reported in terms of all-cause mortality, stroke, and MI. Clinical diversity (e.g., variability in the participant characteristics) and methodological diversity (e.g., different risk of bias sources and varied follow-up durations) among the included studies are possible causes of this observed heterogeneity. However, we addressed this heterogeneity by applying the random-effects model in the analysis and conducting subgroup analyses. Third, potential publication bias was observed in all relevant outcomes. Fourth, data on relevant outcomes (except for all-cause mortality) were not sufficient for further subgroup analyses. Fifth, none of the included studies reported data on any of the relevant outcomes stratified by the participants’ baseline CVD risk score except the study by Gitsels et al. [27]; they grouped the included participants according to the QRISK2 score. Sixth, data on statins safety outcomes (especially SAMS) were not sufficiently reported in the included studies. Seventh, data on nutraceuticals and special diets that may affect the participants’ lipid profile were not reported [89]. Eighth, potential confounders included for the risk estimates adjustment differed among the eligible studies. Finally, this study protocol was not prospectively registered. Most of these limitations reflected on the evidence level, which was rated as “very low” by the GRADE approach. As a result, making clinical recommendations based on the current evidence level is limited. However, our study is complementary to the available evidence from RCTs and can inform future research.

Future studies should employ a randomized design with long-term follow-up periods. A consensus set of standardized outcomes should be provided to and followed by future trialists. The development and validation of a risk score for older people to predict the risk of cardiovascular events can inform the clinical applicability of statin use in this population. In addition, safety outcomes should be a culprit in future studies in this high-risk population, and outcomes that relate to cost-effectiveness and quality of life should also be considered.

## Conclusions

In conclusion, statin therapy appears to be associated with a significantly lower risk of all-cause mortality, CVD death, and stroke (by 14%, 20%, and 15% respectively) in older people aged ≥ 65 years and without CVD. The beneficial association of statins with the risk of all-cause mortality remained significant even at different higher ages. It also was significant in both men and women. However, the association with all-cause mortality remained significant only in older people with DM but not in those without DM. There was no association between statin use and the risk of MI, incident T2DM, or new-onset cancer. These findings suggest statins may offer benefits in the older people in primary prevention setting especially those at the higher risk of CVD (i.e., with DM). As such, these findings support the need for ongoing trials of statins in older adults.

## Supplementary Information

**Additional file 1: Supplementary Figure 1.** Forest plot displaying the results of the meta-analysis of observational studies that compared statin use with non-use in older people aged ≥65 years and without cardiovascular disease – A: in terms of type 2 diabetes mellitus and B: in terms of new-onset cancer. HR, hazard ratio; CI, confidence interval; T2DM, type 2 diabetes mellitus.

**Additional file 2: Supplementary Figure 2.** The corrected funnel plots displaying publication bias in the observational studies that compared statin using with non-using in older people aged ≥65 years and without cardiovascular disease – A: in terms of all-cause mortality; B: in terms of cardiovascular death; C: in terms of myocardial infarction; D: in terms of stroke; E: in terms of type 2 diabetes mellitus and F: in terms of new-onset cancer.

**Additional file 3: Supplementary Table 1.** MOOSE Checklist for Meta-analyses of Observational Studies.

**Additional file 4: Supplementary Table 2.** Literature search strategy for each relevant database.

**Additional file 5: Supplementary Table 3.** List of excluded studies and reasons for exclusion.

**Additional file 6: Supplementary Table 4.** Covariates adjustment in the included studies.

**Additional file 7: Supplementary Table 5.** Risk of bias of included studies using Risk of Bias in Non-randomized Studies of Interventions (ROBINS-I) Tool.

**Additional file 8: Supplementary Table 6.** GRADE assessment of quality of evidence.

**Additional file 9: Supplementary Table 7.** Results of publication bias assessment using funnel plot asymmetry, trim and fill method, and Egger’s test

## Data Availability

The datasets used and/or analyzed during the current study are available from the corresponding author on reasonable request.
