# Supplementary figures and images for: Association of statin use in older people primary prevention group with risk of cardiovascular events and mortality: a systematic review and meta-analysis of observational studies
Source: BMC Med. 2021 Jun 22;19:139. doi: 10.1186/s12916-021-02009-1 (PMC8218529; doi:10.1186/s12916-021-02009-1)

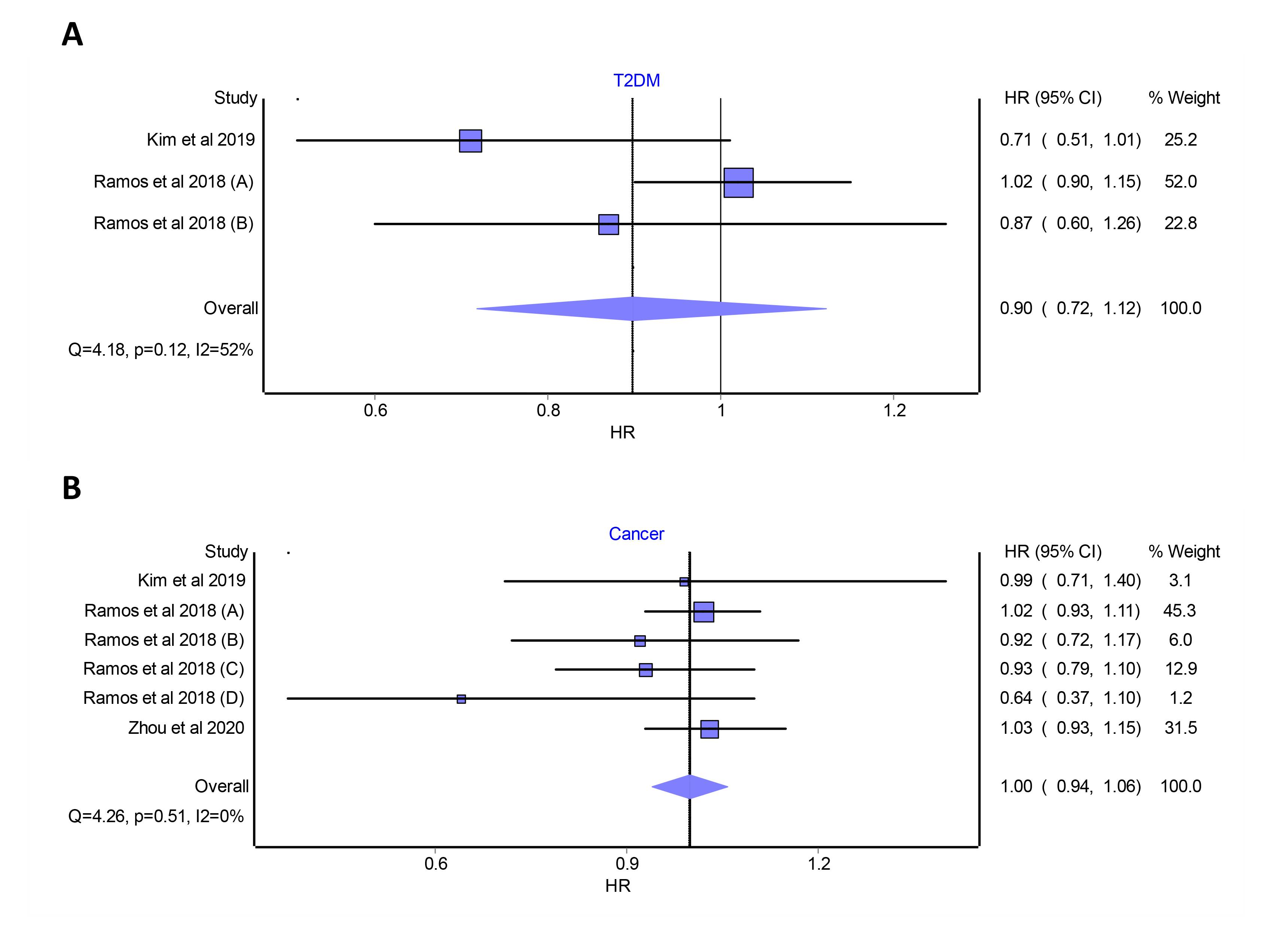

Supplement: Supplementary file 1 — Additional file 1: Supplementary Figure 1. Forest plot displaying the results of the meta-analysis of observational studies that compared statin use with non-use in older people aged ≥65 years and without cardiovascular disease – A: in terms of type 2 diabetes mellitus and B: in terms of new-onset cancer. HR, hazard ratio; CI, confidence interval; T2DM, type 2 diabetes mellitus. [file 12916_2021_2009_MOESM1_ESM.tif]

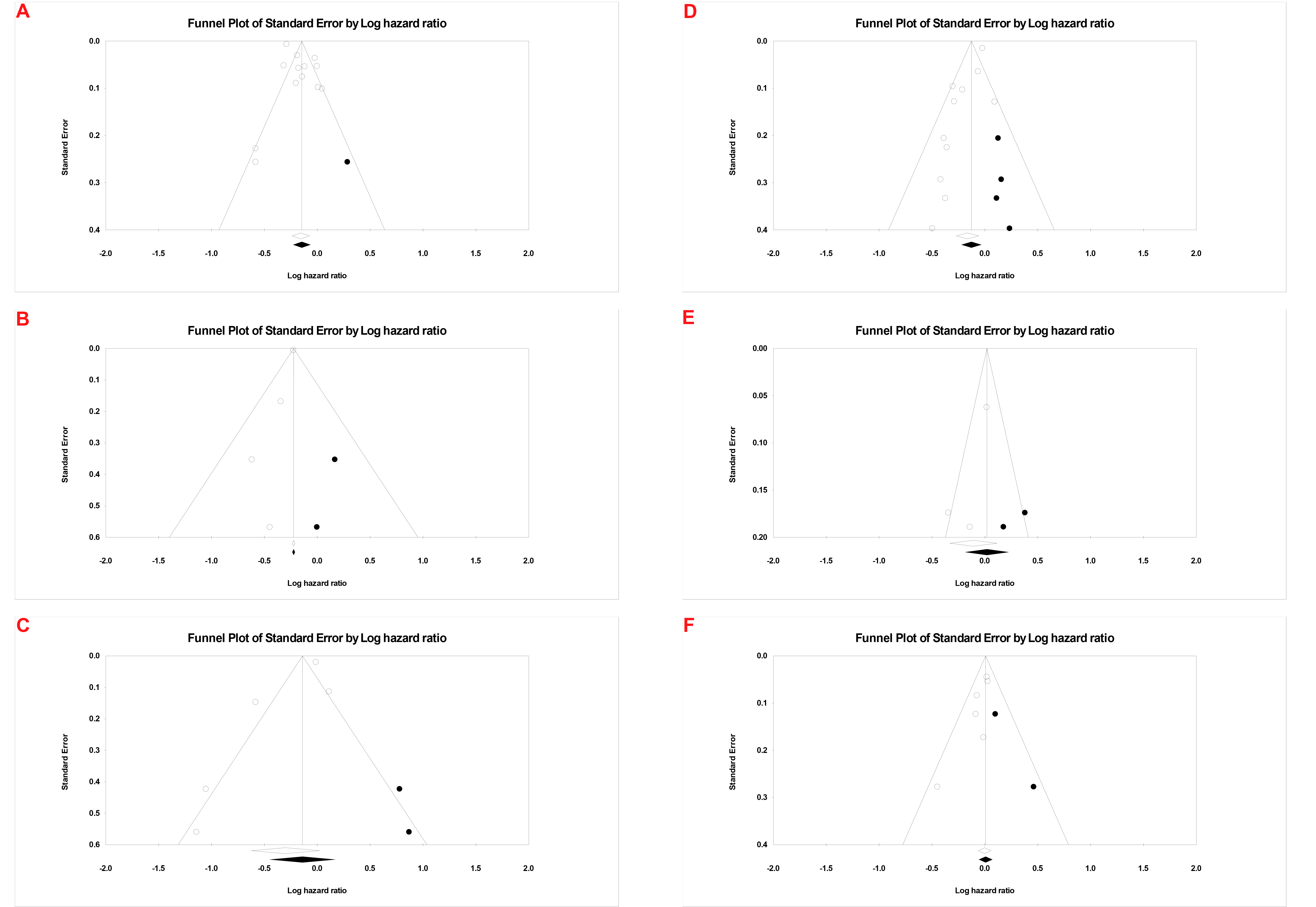

Supplement: Supplementary file 2 — Additional file 2: Supplementary Figure 2. The corrected funnel plots displaying publication bias in the observational studies that compared statin using with non-using in older people aged ≥65 years and without cardiovascular disease – A: in terms of all-cause mortality; B: in terms of cardiovascular death; C: in terms of myocardial infarction; D: in terms of stroke; E: in terms of type 2 diabetes mellitus and F: in terms of new-onset cancer. [file 12916_2021_2009_MOESM2_ESM.tif]
