# Supplementary material for: Association of statin use in older people primary prevention group with risk of cardiovascular events and mortality: a systematic review and meta-analysis of observational studies
Source: BMC Med. 2021 Jun 22;19:139. doi: 10.1186/s12916-021-02009-1 (PMC8218529; doi:10.1186/s12916-021-02009-1)
Supplement: Supplementary file 4 — Additional file 4: Supplementary Table 2. Literature search strategy for each relevant database. [file 12916_2021_2009_MOESM4_ESM.docx]

**Supplementary table 2**: Literature search strategy for each relevant database

| **Database** | **Search keywords** |
| --- | --- |
| PubMed | ("Aged, 80 and over"[Mesh] OR "Aged"[Mesh] OR elderly OR older) AND (atorvastatin OR fluvastatin OR lovastatin OR pitavastatin OR pravastatin OR rosuvastatin OR simvastatin OR lipitor OR zocor OR mevacor OR altoprev OR crestor OR lescol OR livalo OR pravachol OR statin OR statins OR "Hydroxymethylglutaryl-CoA Reductase Inhibitors"[Mesh]) AND (primary) |
| Scopus | (elderly OR older) AND (atorvastatin OR fluvastatin OR lovastatin OR pitavastatin OR pravastatin OR rosuvastatin OR simvastatin OR lipitor OR zocor OR mevacor OR altoprev OR crestor OR lescol OR livalo OR pravachol OR statin OR statins) AND (primary) |
| Embase | (elderly OR older) AND (atorvastatin OR fluvastatin OR lovastatin OR pitavastatin OR pravastatin OR rosuvastatin OR simvastatin OR lipitor OR zocor OR mevacor OR altoprev OR crestor OR lescol OR livalo OR pravachol OR statin OR statins) AND (primary) |
