# Supplementary material for: Association of statin use in older people primary prevention group with risk of cardiovascular events and mortality: a systematic review and meta-analysis of observational studies
Source: BMC Med. 2021 Jun 22;19:139. doi: 10.1186/s12916-021-02009-1 (PMC8218529; doi:10.1186/s12916-021-02009-1)
Supplement: Supplementary file 5 — Additional file 5: Supplementary Table 3. List of excluded studies and reasons for exclusion. [file 12916_2021_2009_MOESM5_ESM.docx]

**Supplementary table 3**: List of excluded studies and reasons for exclusion

| **Study** | **Reason for exclusion** |
| --- | --- |
| Besseling et al. [1] | No data on elderly participants |
| Coste et al. [2] | No relevant outcomes |
| Deambrosis et al. [3] | No data on elderly participants |
| Fung et al. [4] | No data on elderly participants |
| Garcia-Gil et al. [5] | No data on elderly participants |
| Garcia-Gil et al. [6] | No data on elderly participants |
| Hero et al. [7] | No data on elderly participants |
| Jung et al. [8] | Secondary prevention only or no data on primary prevention |
| Motsko et al. [9] | Compared different types of statins |
| Rannanheimo et al. [10] | Assessed the effect of statin adherence degree |
| Sasso et al. [11] | No data on elderly participants |
| Shalev et al. [12] | Assessed the effect of statin adherence degree |
| Shalev et al. [13] | Assessed the effect of statin adherence degree |
| Sheng et al. [14] | No data on elderly participants |
| Blackburn et al. [15] | Contained overlapped data from the same database |
| Eilat-Tsanani et al. [16] | Assessed the effect of statin adherence degree |
| Kim et al. [17] | Compared different types of statins |
| Lang et al. [18] | No data on elderly participants |
| Lee et al. [19] | Used other lipid lowering treatment with statins |
| Versmissen et al. [20] | No data on elderly participants |
| Shah et al. [21] | Secondary prevention only or no data on primary prevention |
| Gnjidic et al. [22] | Secondary prevention only or no data on primary prevention |
| Smeeth et al. [23] | Contained overlapped data from the same database |
| Huesch et al. [24] | Relevant risk estimate was not reported as hazard ratio or odds ratio |
| Kim et al. [25] | Relevant confidence intervals were not reported |
| Pilotto et al. [26] | Secondary prevention only or no data on primary prevention |
| Jacobs et al. [27] | Secondary prevention only or no data on primary prevention |
| Lee et al. [28] | No data on elderly participants |
| Mansi et al. [29] | No data on elderly participants |
| Mansi et al. [30] | No data on elderly participants |
| Sirois et al. [31] | No relevant outcomes |
| Olafsdottir et al. [32] | Participants were free of coronary heart disease with no information about other cardiovascular diseases |
| Palomo-Rodríguez et al. [33] | Non-English content |

**References:**

1. Besseling J, Hovingh GK, Huijgen R, Kastelein JJP, Hutten BA. Statins in Familial Hypercholesterolemia: Consequences for Coronary Artery Disease and All-Cause Mortality. J Am Coll Cardiol. 2016;68:252–60. doi:10.1016/j.jacc.2016.04.054.

2. Coste J, Karras A, Rudnichi A, Dray-Spira R, Pouchot J, Giral P, et al. Statins for primary prevention of cardiovascular disease and the risk of acute kidney injury. Pharmacoepidemiol Drug Saf. 2019;28:1583–90. doi:10.1002/pds.4898.

3. Deambrosis P, Terrazzani G, Walley T, Bader G, Giusti P, Debetto P, et al. Benefit of statins in daily practice? A six-year retrospective observational study. Pharmacol Res. 2009;60:397–401. doi:10.1016/j.phrs.2009.06.009.

4. Fung CSC, Wan EYF, Chan AKC, Lam CLK. Statin use reduces cardiovascular events and all-cause mortality amongst Chinese patients with type 2 diabetes mellitus: A 5-year cohort study. BMC Cardiovasc Disord. 2017;17. doi:10.1186/s12872-017-0599-x.

5. Garcia-Gil M, Comas-Cufí M, Blanch J, Martí R, Ponjoan A, Alves-Cabratosa L, et al. Effectiveness of Statins as Primary Prevention in People With Different Cardiovascular Risk: A Population-Based Cohort Study. Clin Pharmacol Ther. 2018;104:719–32. doi:10.1002/cpt.954.

6. Garcia-Gil M, Comas-Cufí M, Ramos R, Martí R, Alves-Cabratosa L, Parramon D, et al. Effectiveness of Statins as Primary Prevention in People With Gout: A Population-Based Cohort Study. J Cardiovasc Pharmacol Ther. 2019;24:542–50. doi:10.1177/1074248419857071.

7. Hero C, Rawshani A, Svensson AM, Franźen S, Eliassonn B, Eeg-Olofsson K, et al. Association Between Use of Lipid-Lowering Therapy and Cardiovascular Diseases and Death in IndividualsWith Type 1 Diabetes. In: Diabetes Care. American Diabetes Association Inc.; 2016. p. 996–1003. doi:10.2337/dc15-2450.

8. Jung M, Lee S. Effects of Statin Therapy on the Risk of Intracerebral Hemorrhage in Korean Patients with Hyperlipidemia. Pharmacotherapy. 2019;39:129–39. doi:10.1002/phar.2211.

9. Motsko SP, Russmann S, Ming EE, Singh VP, Vendiola RM, Jones JK. Effectiveness of rosuvastatin compared to other statins for the prevention of cardiovascular events - A cohort study in 395 039 patients from clinical practice. Pharmacoepidemiol Drug Saf. 2009;18:1214–22. doi:10.1002/pds.1843.

10. Rannanheimo PK, Tiittanen P, Hartikainen J, Helin-Salmivaara A, Huupponen R, Vahtera J, et al. Impact of Statin Adherence on Cardiovascular Morbidity and All-Cause Mortality in the Primary Prevention of Cardiovascular Disease: A Population-Based Cohort Study in Finland. Value Health. 2015;18:896–905. doi:10.1016/j.jval.2015.06.002.

11. Sasso FC, Lascar N, Ascione A, Carbonara O, De Nicola L, Minutolo R, et al. Moderate-intensity statin therapy seems ineffective in primary cardiovascular prevention in patients with type 2 diabetes complicated by nephropathy. A multicenter prospective 8 years follow up study. Cardiovasc Diabetol. 2016;15. doi:10.1186/s12933-016-0463-9.

12. Shalev V, Chodick G, Silber H, Kokia E, Jan J, Heymann AD. Continuation of statin treatment and all-cause mortality: a population-based cohort study. Arch Intern Med. 2009;169:260–8. doi:10.1001/archinternmed.2008.552.

13. Shalev V, Goldshtein I, Porath A, Weitzman D, Shemer J, Chodick G. Continuation of statin therapy and primary prevention of nonfatal cardiovascular events. Am J Cardiol. 2012;110:1779–86. doi:10.1016/j.amjcard.2012.08.013.

14. Sheng X, Murphy MJ, MacDonald TM, Wei L. Effect of statins on total cholesterol concentrations and cardiovascular outcomes in patients with diabetes mellitus: A population-based cohort study. Eur J Clin Pharmacol. 2012;68:1201–8. doi:10.1007/s00228-012-1234-5.

15. Blackburn R, Osborn D, Walters K, Falcaro M, Nazareth I, Petersen I. Statin prescribing for people with severe mental illnesses: A staggered cohort study of “real-world” impacts. BMJ Open. 2017;7. doi:10.1136/bmjopen-2016-013154.

16. Eilat-Tsanani S, Mor E, Schonmann Y. Statin Use Over 65 Years of Age and All-Cause Mortality: A 10-Year Follow-Up of 19 518 People. J Am Geriatr Soc. 2019;67:2038–44. doi:10.1111/jgs.16060.

17. Kim K, Kwak A, Choi CU, Kim JH, Kim MG, Oh JM, et al. Differences in preventing new-onset cardiovascular events with statin therapy in seniors aged 75 years and over: A cohort study in the South Korean National Health Insurance Database. Basic Clin Pharmacol Toxicol. 2019;125:108–16. doi:10.1111/bcpt.13229.

18. Lang S, Lacombe JM, Mary-Krause M, Partisani M, Bidegain F, Cotte L, et al. Is impact of statin therapy on all-cause mortality different in HIV-infected individuals compared to general population? Results from the FHDH-ANRS CO4 cohort. PLoS One. 2015;10. doi:10.1371/journal.pone.0133358.

19. Lee VWY, Ho ICH, Chan WSY, Tam KY, Lee KKC. Statin utilization patterns for the primary prevention of cardiovascular events: A retrospective study in patients with diabetes mellitus in Hong Kong. Am J Cardiovasc Drugs. 2008;8:199–205. doi:10.2165/00129784-200808030-00006.

20. Versmissen J, Oosterveer DM, Yazdanpanah M, Defesche JC, Basart DCG, Liem AH, et al. Efficacy of statins in familial hypercholesterolaemia: A long term cohort study. BMJ. 2009;338:223–6. doi:10.1136/bmj.a2423.

21. Shah R, Wang Y, Foody JAM. Effect of Statins, Angiotensin-Converting Enzyme Inhibitors, and Beta Blockers on Survival in Patients ≥65 Years of Age With Heart Failure and Preserved Left Ventricular Systolic Function. Am J Cardiol. 2008;101:217–22. doi:10.1016/j.amjcard.2007.08.050.

22. Gnjidic D, Le Couteur DG, Blyth FM, Travison T, Rogers K, Naganathan V, et al. Statin use and clinical outcomes in older men: A prospective population-based study. BMJ Open. 2013;3. doi:10.1136/bmjopen-2012-002333.

23. Smeeth L, Douglas I, Hall AJ, Hubbard R, Evans S. Effect of statins on a wide range of health outcomes: A cohort study validated by comparison with randomized trials. Br J Clin Pharmacol. 2009;67:99–109. doi:10.1111/j.1365-2125.2008.03308.x.

24. Huesch MD. Association of baseline statin use among older adults without clinical cardiovascular disease in the SPRINT trial. JAMA Internal Medicine. 2018;178:560–1. doi:10.1001/jamainternmed.2017.7844.

25. Kim S, Choi H, Won CW. Effects of statin use for primary prevention among adults aged 75 years and older in the National Health Insurance Service senior cohort (2002– 2015). Ann Geriatr Med Res. 2020;24:91–8. doi:10.4235/AGMR.20.0028.

26. Pilotto A, Panza F, Copetti M, Simonato M, Sancarlo D, Gallina P, et al. Statin treatment and mortality in community-dwelling frail older patients with diabetes mellitus: A retrospective observational study. PLoS One. 2015;10. doi:10.1371/journal.pone.0130946.

27. Jacobs JM, Cohen A, Ein-Mor E, Stessman J. Cholesterol, statins, and longevity from age 70 to 90 years. J Am Med Dir Assoc. 2013;14:883–8. doi:10.1016/j.jamda.2013.08.012.

28. Lee CJ, Oh J, Lee SH, Kang SM, Choi D, Kim HC, et al. Efficacy of aspirin and statins in primary prevention of cardiovascularmortality in uncomplicated hypertensive participants: A Korean national cohort study. J Hypertens. 2017;35:S33–40. doi:10.1097/HJH.0000000000001279.

29. Mansi IA, English J, Zhang S, Mortensen EM, Halm EA. Long-Term Outcomes of Short-Term Statin Use in Healthy Adults: A Retrospective Cohort Study. Drug Saf. 2016;39:543–59. doi:10.1007/s40264-016-0412-2.

30. Mansi IA, English JL, Morris MJ, Zhang S, Mortensen EM, Halm EA. Statins for primary prevention in physically active individuals: Do the risks outweigh the benefits? J Sci Med Sport. 2017;20:627–32. doi:10.1016/j.jsams.2016.12.075.

31. Sirois C, Moisan J, Poirier P, Grégoire JP. Comparative effectiveness of cardioprotective drugs in elderly individuals with type 2 diabetes. Int J Clin Pract. 2015;69:305–12. doi:10.1111/ijcp.12503.

32. Olafsdottir E, Aspelund T, Sigurdsson G, Thorsson B, Eiriksdottir G, Harris TB, et al. Effects of statin medication on mortality risk associated with type 2 diabetes in older persons: the population-based AGES-Reykjavik Study. BMJ Open. 2011;1:e000132–e000132. doi:10.1136/bmjopen-2011-000132.

33. Palomo-Rodríguez R, Ortega-Blanco JA, Pedregal-González M, Serrano-Nogales R. Statin treatment as primary prevention in dyslipidaemic patients older than 75 years. Semergen. 2020. doi:10.1016/j.semerg.2020.04.006.
