# Supplementary material for: Association of statin use in older people primary prevention group with risk of cardiovascular events and mortality: a systematic review and meta-analysis of observational studies
Source: BMC Med. 2021 Jun 22;19:139. doi: 10.1186/s12916-021-02009-1 (PMC8218529; doi:10.1186/s12916-021-02009-1)
Supplement: Supplementary file 6 — Additional file 6: Supplementary Table 4. Covariates adjustment in the included studies. [file 12916_2021_2009_MOESM6_ESM.docx]

**Supplementary table 4:** Covariates adjustment in the included studies

| Study | Covariates adjustment |
| --- | --- |
| Alpérovitch et al. 2015 | Sex, center, DM, BMI, alcohol use, smoking, HTN, cardiac rhythm disorder, antithrombotic therapy, TG, and LDL-C/HDL-C ratio |
| Bezin et al. 2019 | Age, sex, abnormal liver function, abnormal renal function, cardiac arrhythmia, COPD, coronary intervention (PCI OR CABG), dementia, DM, HF, major hemorrhage, neoplasm, peripheral artery disease, psychiatric disorder, stroke, transient ischemic attack, anticoagulant drugs, antidepressant drugs, antiplatelet agents, beta-blockers, CCBs, diuretics, drugs acting on RAAS, nitrates, other antihypertensive drugs, and other LLTs |
| Gitsels et al. 2016 | Sex, year of birth, socioeconomic status, DM, hypercholesterolemia, blood pressure regulating drugs, BMI, smoking status, and general practice |
| Jun et al. 2019 | Age, sex, income category, DM, HTN, and previous use of other LLTs |
| Kim et al. 2019 | Age, sex, HTN, DM, chronic thyroid disease, chronic pulmonary disease, congestive HF, AF, renal insufficiency, BMI, baseline lipid profiles, and current medications (anticoagulant, antiplatelet, and number of antihypertensive agents) |
| Lemaitre al. 2002 | Age, sex, DM, and prevalent CVD (angina, CABG, angioplasty, carotid endarterectomy, or bypass procedure on a leg artery). |
| Orkaby et al. 2017 | Age, BMI, white race, smoking status, hyperlipidemia, HTN, DM, HF, renal disease, liver disease, cancer, depression, dementia, aspirin use, HTN medication, DM medication, alcohol use, and health significantly limits |
| Orkaby et al. 2020 | Age, sex, race, ethnicity, BMI, region of the country, hyperlipidemia, HTN, DM, liver disease, other LLTs, smoking status, anemia, cancer, HF, AF, CKD, substance abuse, mental health disorders, arthritis, dementia, polypharmacy, fatigue and gait abnormality |
| Ramos et al. 2018 | Age, sex, height, weight, systolic and diastolic blood pressure, Medea deprivation index, smoking, glucose, total cholesterol, LDL-C, HDL-C, obesity, hypercholesterolemia, valvular heart disease, AF, benign neoplasm, HTN, asthma, COPD, sleep apnea, arthritis, hyperthyroidism, hypothyroidism, CKD, diuretics, beta blocking agents, CCB, agents acting on RAAS, other antihypertensives, antidiabetic drugs, corticosteroids for systemic use, anti-inflammatory and antirheumatic drugs, psycholeptics, and psychoanaleptics |
| Zhou et al. 2020 | Age, sex, ethnicity/race, smoking status, alcohol use, BMI, family history of CVD, history of cancer, heart rate, DM, CKD, HTN, living alone, education, gait time to walk 3 meters, grip strength of dominant hand, number of total concomitant medications, other LLTs, antihypertensive agents, oral hypoglycemic agents, insulin, and previous regular aspirin use |

Abbreviations: DM diabetes mellites; BMI body mass index; HTN hypertension; TG triglycerides; LDL-C low-density lipoprotein cholesterol; HDL-C high-density lipoprotein cholesterol; COPD chronic obstructive pulmonary disease; PCI percutaneous coronary intervention; CABG coronary artery bypass graft; HF heart failure; CCBs calcium channel blockers; RAAS renin-angiotensin-aldosterone system; LLTs lipid lowering treatments; AF atrial fibrillation; CVD cardiovascular disease; CKD chronic kidney disease
