# Supplementary material for: Association of statin use in older people primary prevention group with risk of cardiovascular events and mortality: a systematic review and meta-analysis of observational studies
Source: BMC Med. 2021 Jun 22;19:139. doi: 10.1186/s12916-021-02009-1 (PMC8218529; doi:10.1186/s12916-021-02009-1)
Supplement: Supplementary file 7 — Additional file 7: Supplementary Table 5. Risk of bias of included studies using Risk of Bias in Non-randomized Studies of Interventions (ROBINS-I) Tool. [file 12916_2021_2009_MOESM7_ESM.docx]

**Supplementary table 5**: Risk of bias of included studies using Risk of Bias in Non-randomized Studies of Interventions (ROBINS-I) Tool

| **Study** | **Bias due to**  **confounding** | **Bias in**  **selection of**  **participants**  **into the study** | **Bias in**  **classification**  **of**  **interventions** | **Bias due to**  **deviations**  **from intended interventions** | **Bias due to**  **missing data** | **Bias in**  **measurement**  **of outcomes** | **Bias in**  **selection of**  **the reported**  **result** | **Overall bias** |
| --- | --- | --- | --- | --- | --- | --- | --- | --- |
| Alpérovitch et al. 2015 | Moderate | Serious | Low | Low | Low | Moderate | Low | Serious |
| Bezin et al. 2019 | Moderate | Low | Low | Low | Low | Low | Low | Moderate |
| Gitsels et al. 2016 | Moderate | Serious | Low | Low | Low | Low | Low | Serious |
| Jun et al. 2019 | Serious | Serious | Moderate | Low | Low | Low | Low | Serious |
| Kim et al. 2019 | Moderate | Low | Low | Low | Low | Moderate | Low | Moderate |
| Lemaitre et al. 2002 | Serious | Serious | Low | Low | Low | Moderate | Low | Serious |
| Orkaby et al. 2017 | Serious | Serious | Moderate | Low | Low | Moderate | Low | Serious |
| Orkaby et al. 2020 | Moderate | Low | Low | Low | Low | Low | Low | Moderate |
| Ramos et al. 2018 | Moderate | Low | Low | Low | Low | Low | Low | Moderate |
| Zhou et al. 2020 | Moderate | Serious | Low | Low | Low | Low | Low | Serious |
