# Supplementary material for: Association of statin use in older people primary prevention group with risk of cardiovascular events and mortality: a systematic review and meta-analysis of observational studies
Source: BMC Med. 2021 Jun 22;19:139. doi: 10.1186/s12916-021-02009-1 (PMC8218529; doi:10.1186/s12916-021-02009-1)
Supplement: Supplementary file 8 — Additional file 8: Supplementary Table 6. GRADE assessment of quality of evidence. [file 12916_2021_2009_MOESM8_ESM.docx]

**Supplementary Table 6**: GRADE assessment of quality of evidence

| **Outcomes** | **Risk of bias^*^** | **Inconsistency** | **Indirectness** | **Imprecision** | **Publication bias** | **Large effect** | **Dose response** | **Residual bias** | **Quality of evidence** |
| --- | --- | --- | --- | --- | --- | --- | --- | --- | --- |
| All-cause mortality | Serious | Very serious^**^ | Not serious | Not serious | Very strongly suspected^††^ | Undetected | Undetected | Undetected | **⊕**⭕⭕⭕  Very low |
| CVD death | Serious | Not serious | Not serious | Not serious | Strongly suspected^‡^ | Undetected | Undetected | Undetected | **⊕**⭕⭕⭕  Very low |
| Stroke | Serious | Serious^***^ | Not serious | Not serious | Very strongly suspected^††^ | Undetected | Undetected | Undetected | **⊕**⭕⭕⭕  Very low |
| MI | Serious | Very serious^**^ | Not serious | Serious^†^ | Strongly suspected^‡^ | Undetected | Undetected | Undetected | **⊕**⭕⭕⭕  Very low |

^*^According to The Risk Of Bias In Non-randomized Studies of Interventions (ROBINS-I) tool

^**^Significant heterogeneity (I^2^>80%, P value<0.0001) and not overlapping confidence intervals

^***^Significant heterogeneity (I^2^=61%, P value<0.0001) and relatively overlapping confidence intervals

^†^Very wide confidence interval

^††^Suggested by both visual inspection of funnel plot asymmetry and Egger`s test

^‡^Suggested only by visual inspection of funnel plot asymmetry
