# Supplementary material for: Association of statin use in older people primary prevention group with risk of cardiovascular events and mortality: a systematic review and meta-analysis of observational studies
Source: BMC Med. 2021 Jun 22;19:139. doi: 10.1186/s12916-021-02009-1 (PMC8218529; doi:10.1186/s12916-021-02009-1)
Supplement: Supplementary file 9 — Additional file 9: Supplementary Table 7. Results of publication bias assessment using funnel plot asymmetry, trim and fill method, and Egger’s test [file 12916_2021_2009_MOESM9_ESM.docx]

**Supplementary table 7**: Results of publication bias assessment using funnel plot asymmetry, trim and fill method, and Egger`s test

| **Outcomes** | **Funnel plot** | **Trim and fill adjusted ES (95% CI)** | | | **Egger`s test^*^** |
| --- | --- | --- | --- | --- | --- |
|  |  | **Before** | **After** | **# of imputed studies** |  |
| All-cause mortality | Asymmetrical | 0.86 (0.79 to 0.93) | 0.86 (0.80 to 0.94) | 1 | **0.026** |
| CVD death | Asymmetrical | 0.80 (0.78 to 0.81) | 0.80 (0.79 to 0.81) | 2 | 0.072 |
| MI | Asymmetrical | 0.74 (0.53 to 1.02) | 0.87 (0.64 to 1.19) | 2 | 0.165 |
| Stroke | Asymmetrical | 0.85 (0.76 to 0.94) | 0.88 (0.80 to 0.97) | 4 | **0.003** |
| T2DM | Asymmetrical | 0.90 (0.72 to 1.12) | 1.02 (0.83 to 1.25) | 2 | 0.297 |
| New-onset cancer | Asymmetrical | 1 (0.94 to 1.06) | 1.01 (0.94 to 1.07) | 2 | **0.042** |

Abbreviations: ES effect estimate; CI confidence interval; CVD cardiovascular disease; T2DM type 2 diabetes mellites.

^*^2-tailed p values
